# How Long Should We Wait to Create the Goutallier Stage 2 Fatty Infiltrations in the Rabbit Shoulder for Repairable Rotator Cuff Tear Model?

**DOI:** 10.1155/2019/7387131

**Published:** 2019-04-02

**Authors:** Mohamed Attia Abdou, Ga-Eon Kim, Jangho Kim, Byung-Hoon Kim, Yang-Kyung Kim, Sung-Eun Jeong, Tae-Jin Kim, Hyeng-Kyu Park, Myung-Sun Kim

**Affiliations:** ^1^Department of Orthopaedics, Chonnam National University, College of Medicine, Gwangju 61649, Republic of Korea; ^2^Department of Biomedical Sciences, Chonnam National University, College of Medicine, Gwangju 61649, Republic of Korea; ^3^Department of Pathology, Chonnam National University, College of Medicine, Gwangju 61649, Republic of Korea; ^4^Department of Rural and Biosystems Engineering, Chonnam National University, Gwangju 61186, Republic of Korea; ^5^Department of Dental Materials, Chosun University, School of Dentistry, MRC, Gwangju 61452, Republic of Korea; ^6^Department of Physical and Rehabilitation Medicine, Chonnam National University, College of Medicine, Gwangju 61469, Republic of Korea

## Abstract

**Background:**

Significant proportion of rotator cuff tears (RCTs) in clinical field are of a kind of repairable tear wherein the degree of fatty infiltration is of Goutallier stage 1 or stage 2. Therefore, the animal model, showing similar fatty infiltration, seems preferable for researches. The purpose of this study is to find out the proper time frame in which there is Goutallier stage 1 or stage 2 fatty infiltration in the rabbit RCT model for the research of repairable RCT in humans.

**Methods:**

Supraspinatus tendon tears were created in forty male New Zealand white rabbits at their right shoulder (n= 8 for each group), and a sham operation on the left shoulder. Rabbits were divided into five groups (2nd, 4th, 6th, 8th, and 12th weeks). Specimens were harvested from the central portion of the supraspinatus muscle for haematoxylin and eosin (H &E) staining, followed by histological and Goutallier grading evaluation. Results are expressed as mean ± standard deviation by Sigma Plot software (version 7.0).

**Results:**

At two weeks, mainly lipoblasts were observed around the muscle fibers, and at four weeks these lipoblasts were replaced by mature adipocytes with fatty infiltration amount (2.13 ± 0.35). The degree of muscle atrophy was (1.50 ± 0.53) at four weeks compared to sham group (0.88 ± 0.64) with significant difference (*p* < 0.05). The inflammatory process appeared as two phases. At two weeks, it was increased with grading value (1.88 ± 0.35). However, in the four-week group, it showed a sharp decrease (0.50 ± 0.53). At six weeks, inflammation reappeared to increase (1.13 ± 0.83). Then, a gradual decline appeared at eight weeks (0.88 ± 0.83) and at 12 weeks (0.50 ± 0.92).

**Conclusions:**

At two and four weeks, both fat distribution in rabbit supraspinatus muscles and Goutallier grading scale mostly appeared as grade 2. Therefore, we can consider four weeks to be a suitable period for making a repairable RCT animal model for the human research, considering the early acute tissue reaction at 2 weeks after the tendon tears.

## 1. Introduction

Rotator cuff tears (RCTs) are a pathological condition that frequently affects aged populations. RCT often causes persistent pain and severe functional impairments in the affected shoulder joint. Although there are advanced surgical techniques, the management of RCTs remains a challenge [1]. The detachment of tendon from bone and the accompanying unloading of tensile forces leads to changes in the structure and function of the tendon and muscle [2, 3].

Fatty infiltration of the rotator cuff was described as adipocytes deposition within the rotator cuff muscles [4]. The degenerated supraspinatus muscle shows fatty infiltration, atrophy, and retraction [5, 6]. The Goutallier classification system for fatty infiltration has become a standard reference for determining the severity of fatty infiltration and uses CT images of the rotator cuff muscle [7].

The fatty degeneration and the atrophy of the RCT muscle is an important factor in the prognosis for the healing of the rotator cuff repair as well as surgical outcome [3, 8, 9]. According to Thomazeau et al. [10], the tendon tear becomes irreparable if there is stage 3 muscle atrophy or, according Goutallier et al., if there is stage 3 or stage 4 fatty infiltration of the muscle [4, 11]. On the other hand, Goutallier stage 1 or stage 2 fatty infiltration may indicate that the tendon tear is repairable. Clinically, surgical repair looks appropriate in most RCT patients with a fat infiltration index smaller than grade 2 [12].

Actually, a significant proportion of RCTs in clinical field is of a kind of repairable tear wherein the degree of fatty infiltration is of stage 1 or stage 2. Therefore, the animal model, showing fatty infiltration grade 1 or grade 2 to rotator cuff muscle, seems preferable for researches to be conducted in association with patients of repairable RCT.

Preclinical animal models are rich tools in the search for understanding of the basics of human pathology at the cellular and tissue levels, as well as for the evaluation of new therapeutics and surgical techniques [13, 14]. Previous studies have used rabbit models for research on rotator cuff diseases. Fabis et al. have described fatty infiltration development in rabbit supraspinatus in relation to the degree of tendon detachment [15, 16]. Some described pathology based on two-week time point [13, 17–19]; others also used four weeks [20–26] or six weeks [27–31] or eight weeks [32, 33] or 12 weeks [29, 31, 34–36].

The purpose of this study is to gain more understanding of the proper time frame in which there is Goutallier stage 1 or stage 2 fatty infiltration in the rabbit RCT model for the research of repairable RCT in humans. Our hypothesis is that the 4-week model demonstrates Goutallier stage 1 or stage 2 fatty infiltration consistent with a repairable model for human RCT research.

## 2. Materials and Methods

All experimental procedures were approved by the experimental animal committee of the biomedical research institute of Chonnam National University (CNU IACUC-H-2016-33).

### 2.1. Rabbit's Allocation

A total of 40 male New Zealand white rabbits (Damul Science, Daejeon, South Korea), aged four months, with a mean body weight of 2.8 kg (range: 2.5 to 3 kg) were used. The rabbits were housed in light within temperature controlled rooms and fed a standard diet. The animals were observed for one week before surgery to confirm that they were healthy and disease-free. The animals were randomly allocated into five groups: 2 weeks (n= 8); 4 weeks (n= 8); 6 weeks (n= 8); 8 weeks (n= 8); 12 weeks (n= 8). Lesion was defined as supraspinatus tendon detachment. The experimental control (sham) at left side was defined as a skin incision and retraction of both omotransversarius and trapezius muscles from scapular spine, without any gesture carried out in the rotator cuff tendons.

### 2.2. Surgical Procedure

All animals were anesthetized by induction with ketamine (35 mg/kg) (Youhan Corporation, Seoul, South Korea) and xylazine (5 mg/kg) in one syringe for each rabbit [36]. Once the rabbits were anesthetized, we shaved around their shoulder joints at the area of the surgical site, the skin was disinfected with povidone-iodine (Firson, South Korea), and the animals were placed in the lateral position with the forelimbs in adduction and external rotation.

**Figure 1:**
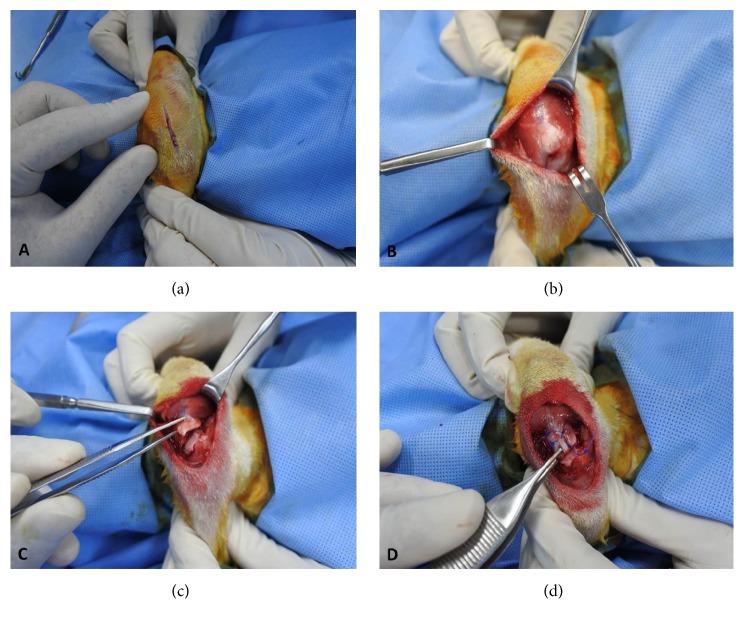
(a) shows the 3.0 cm skin incision at the glenohumeral joint, (b) the exposed supraspinatus tendon at the foot print, (c) the large detached supraspinatus tendon from the greater tuberosity, and (d) wrapped the silicone Penrose drain around the detached supraspinatus tendon.

A 3.0 cm incision was made in the skin over the glenohumeral joint. The subcutaneous tissue was dissected; retraction of omotransversarius and trapezius muscles was performed to expose the supraspinatus tendon (located in a superior position of the scapular spine) [37] (Figures 1(a) and 1(b)).

As described by Fabis et al. [15], sharp release of supraspinatus tendon was performed at the greater tuberosity of the humerus (large detachment) as well as from the surrounding subscapularis and infraspinatus tendons (cut interdigitation-length of split about 1 cm) (Figure 1(c)). Then the detached tendon stump was wrapped with a silicone Penrose drain, 10 mm long (8mm in outer diameter, Yushin Corp, Bucheon, South Korea), to prevent adhesion to the surroundings (Figure 1(d)) [38]. The wounds were then irrigated and closed in layers with Vicryl® 4-0 (Ethicon, Johnson and Johnson, USA). Meanwhile, for the control group, the sham operation was done on the left side, without release of the supraspinatus tendon after retraction of the omotransversarius and trapezius muscles. All rabbits tolerated this procedure without any intraoperative complications.

After surgery, analgesia (meloxicam 1 mg/kg), and antibiotic (enrofloxacin, 1 mg/ml, Bayer, Germany), intramuscular injections were administered to control pain and infection, respectively. Postoperative immobilization was not prescribed. Rabbits were kept in separate stainless steel cages in the animal house and allowed free access to both food which was supplied as a compound pellets by Cargill Agri Purina, Inc. Kunsan Plant, South Korea, and water.

### 2.3. Collection of Specimens

**Figure 2:**
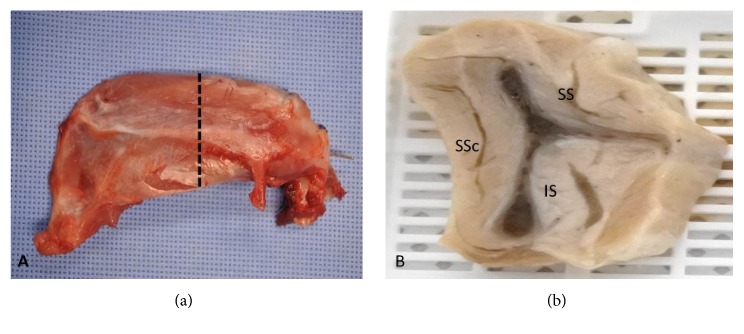
(a) shows the scapula with the whole surrounding rotator cuff muscles, posterior view; dashed line shows the cutting site at the supraspinatus central portion, (b) the scapular Y view portion after central cutting, shows supraspinatus muscle (SS), infraspinatus muscle (IS), and subscapularis muscle (SSc).

The 40 rabbits were randomly euthanized at the second, fourth, sixth, eighth, and 12th weeks after surgery, so that a histological assessment could be performed. A lethal dose of intravenous KCL (5ml) was injected after induction of intramuscular anaesthesia, and the rabbits entered a deep sleep. The scapula with the attached four rotator cuff muscles and the proximal half of the humerus were removed en bloc after dissection from both shoulders of each rabbit (Figure 2(a)).

### 2.4. Histological Evaluation

The specimens were immediately fixed in neutral buffered 10% formalin (PH 7.4) and maintained thus for two days. Then they were decalcified with histological decalcifying agent HCL (Calci-clear Rapid, National Diagnostics 305 Patton Drive Atlanta, GA 30036, USA) for 10 days. Residual decalcifying agent was removed by immersion in tap water for 24 hr.

In each rabbit, the supraspinatus muscle was sectioned in the transverse (sagittal) plane at 10 *μ*m thickness from the center (Figure 2(b)) and processed with paraffin embedding. Four *μ*m sections were cut on a rotary microtome, mounted on glass slides, and dried overnight at 45°C. The slides were stained with haematoxylin and eosin (H&E), and then the slides were scanned and read by the Aperio image analysis system [V 12.3.2.8013] (Leica Biosystems, Vista, USA) and light microscopy.

Based on a four-stage scale, in a manner consistent with the Goutallier classification for fatty infiltrations of the supraspinatus muscles in each group, the H&E staining allowed a semiquantitative assessment of the amount of fat infiltration within the affected muscle. Stage 0 = a completely normal muscle; stage 1 = muscle contains some fatty streaks; stage 2 = fatty infiltration is still less than muscle; stage 3 = there is as much muscle as fat; stage 4 = more fat than muscle [39, 40].

In addition, H&E stained slides were graded semiquantitatively for the muscle atrophy as follows: normal =0, mild atrophy =1, moderate=2, and severe =3. These grades were identified based on a few suggestive findings, such as an angular shape of muscle fibers as opposed to a round shape, the decreased muscle fiber size, and decreased distance between myonuclei and centralized myonuclei [40, 41]. Also, inflammatory cells was graded as follows: 0 = no inflammatory cell, 1 = mild, 2 = moderate, and 3 = severe [40, 41].

### 2.5. Statistical Analysis

Results are expressed as mean ± standard deviation by Sigma Plot software 2001 (version 7.0). Differences between each group were regarded as statistically significant when P < 0.05.

## 3. Results

### 3.1. Gross Observation at Dissection

**Figure 3:**
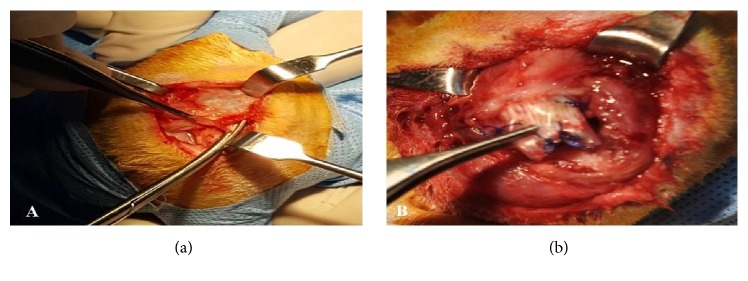
Shows the gross findings during the dissection. (a) The scarring tissue covering the supraspinatus tendon; (b) the supraspinatus tendon stump, wrapped by silicone Penrose drain.

All detached tendon stumps were found to be adhered around the glenoid, and they were retracted medially at the time of dissection. We observed bursal tissue thickening, and, in all groups, fibrous tissue was filling the gap between the greater tuberosity and the retracted tendon; we removed the scar tissue to find out the wrapped Penrose drain around the detached tendon (Figure 3). The supraspinatus muscle was observed as a smaller than the control muscle on the contralateral side. In the sham operated group, there was no evidence of either muscle atrophy or adhesions.

### 3.2. Pattern of Fatty Infiltration

**Figure 4:**
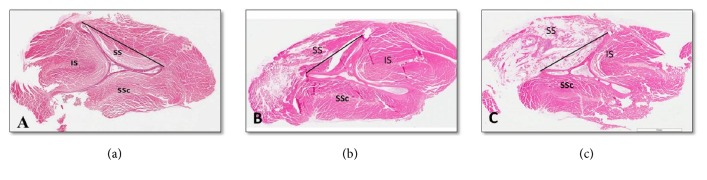
Fatty infiltration distribution in the middle part of supraspinatus muscles of experimental groups (scale bar: 7 *μ*m). (a) Below tangent line, (b) above the tangent line, and (c) both below and above the tangent line.

The majority of samples were noticed to have fatty infiltration distribution below the tangent line among the all 40 samples (Figure 4), in which* 8/8 at 2wks, 7/8 at 4wks, 6/8 at 6wks, 5/8 at 8wks, and 1/8 at 12 wks* showed the below tangent line pattern Table 1.

### 3.3. Grading of Fatty Infiltration

**Figure 5:**
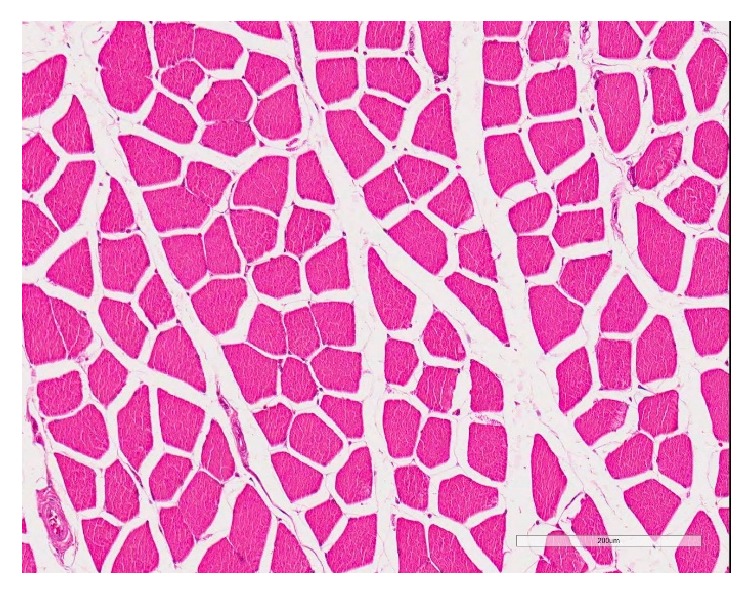
The supraspinatus muscle (H&E) in the sham group with almost normal muscle fibers, interfascicular fat is nil [scale bar 200 *μ*m].

Histological analysis demonstrated more obvious morphologic changes in the transected supraspinatus muscles than sham group which appeared almost normal with nil interfascicular fat (Figure 5). For most of the all operated groups, adipocytes were observed in clusters among the supraspinatus muscle fascicles with a characteristic signet ring appearance, and they were more numerous than in the controls (Table 2). Semiquantitative analysis of H&E stained specimens showed significant higher fat contents in the supraspinatus muscle after the tendon tear (Table 3). Interclass correlation coefficients for the three raters were >0.7 for the evaluation of fat infiltration, muscle atrophy, and inflammation (Table 4).

**Figure 6:**
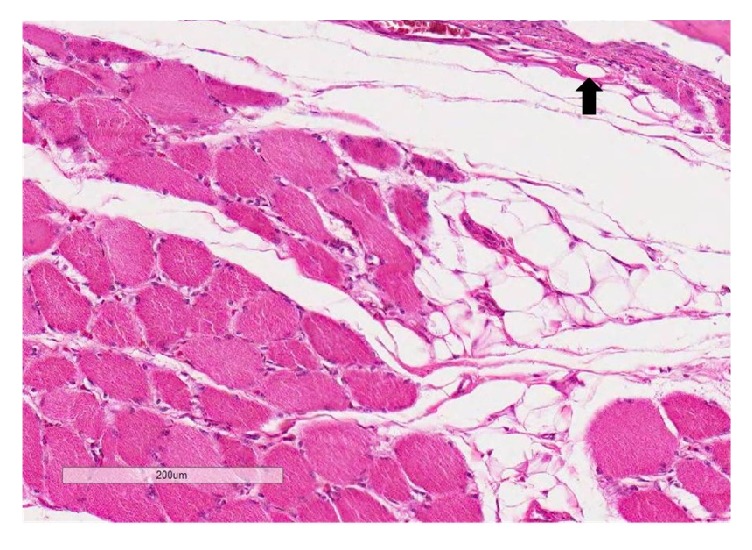
The supraspinatus muscle section (H&E) showing a grade 2 fatty infiltration at 2 weeks [scale bar 200 *μ*m]. There is infiltration of lipoblasts (black arrow) and inflammatory cells around muscle fibers.

**Figure 7:**
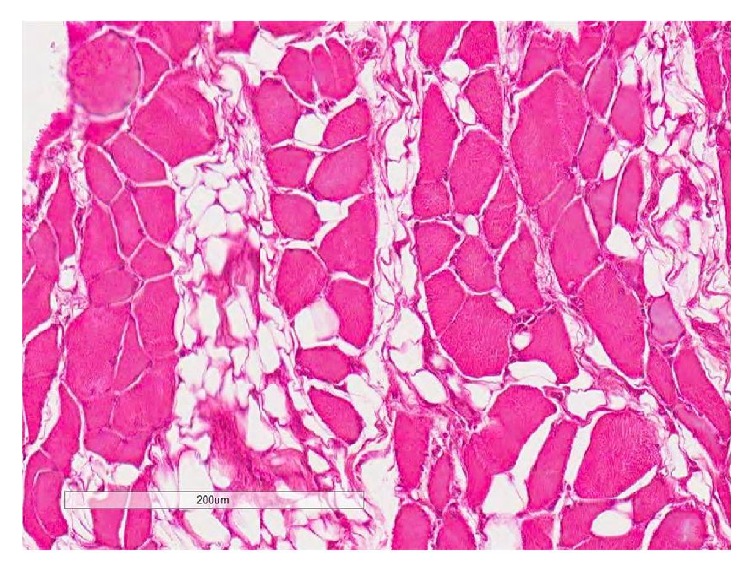
The supraspinatus muscle section (H&E) showing adipocytes around muscle fibers at 4 weeks with fatty infiltration appearing as grade 2.

**Figure 8:**
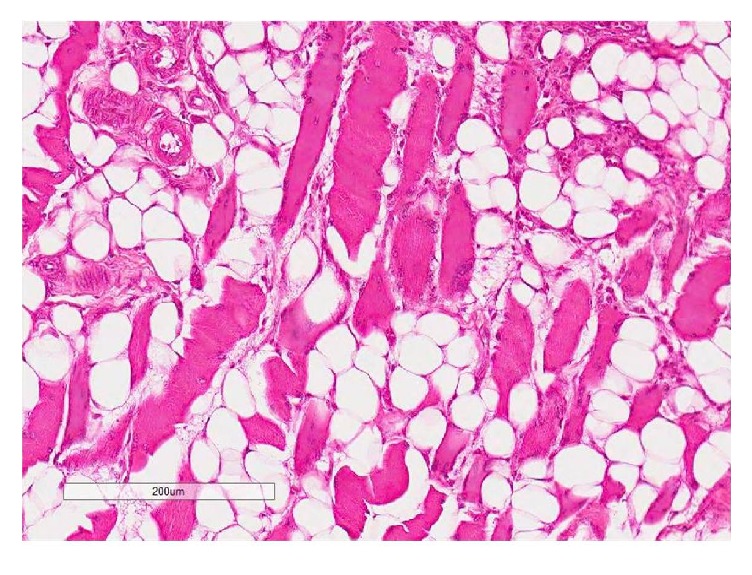
The supraspinatus muscle section (H&E) demonstrating adipocytes around muscle fibers at 6 weeks with fatty infiltration appearing as grade 3.

**Figure 9:**
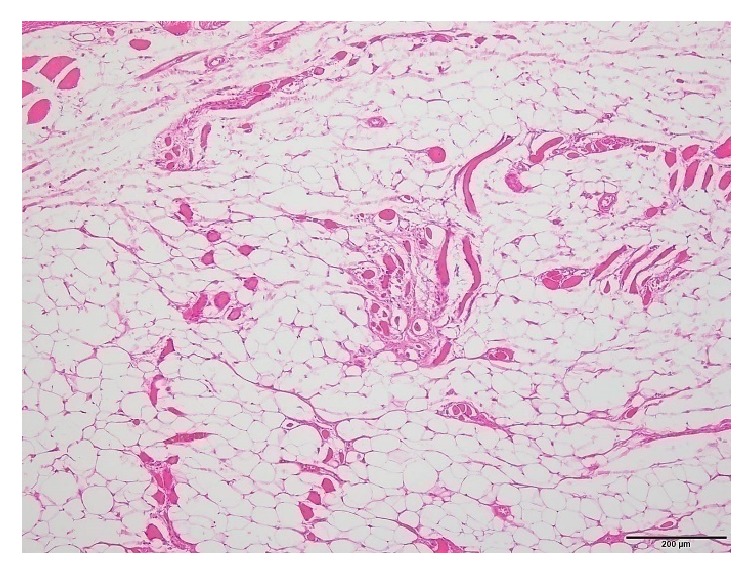
The supraspinatus muscle section (H&E) illustrating adipocytes around muscle fibers at 8 weeks with fatty infiltration appearing as grade 4.

**Figure 10:**
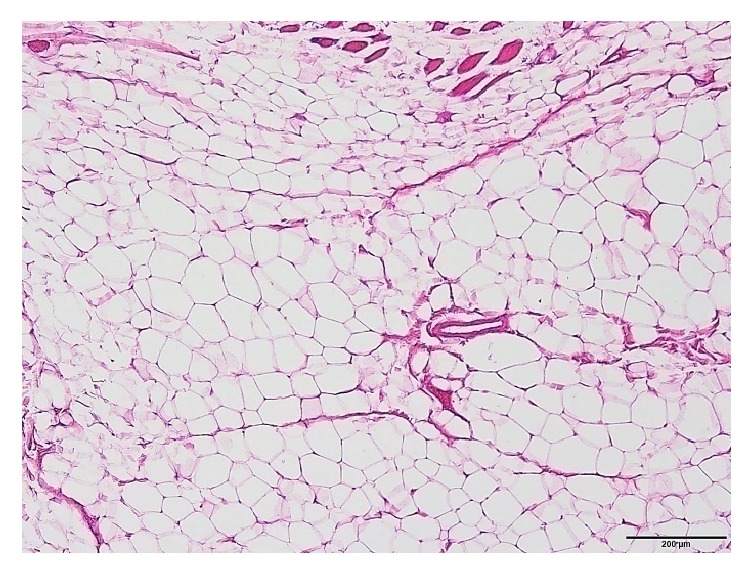
The supraspinatus muscle section (H&E) shows extensive fatty infiltration with a few scattered muscle fibers (grade 4) at 12 weeks.

**Figure 11:**
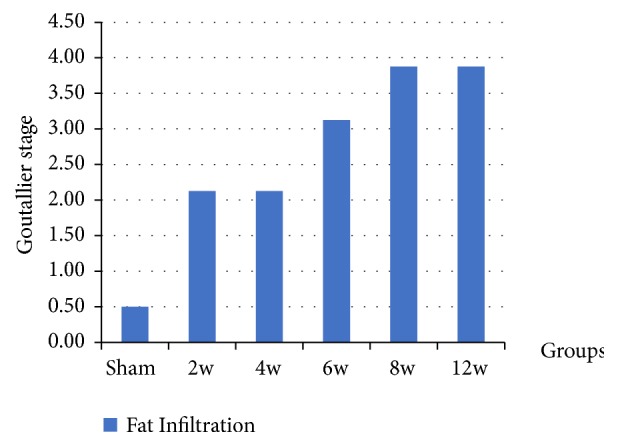
The graph shows the fatty infiltration grading and the data represented by mean ± SD.

At two weeks, mainly immature adipocytes (lipoblasts) were observed around the muscle fibers (Figure 6), and at four weeks these lipoblasts were replaced by mature adipocytes (Figure 7). The average fat grading at two weeks was (2.13 ± 0.64), and at four weeks it was (2.13 ± 0.35). This is corresponding mostly to grade 2 on the Goutallier scale. At six weeks, the average grading of the adipocytes between muscle fibers was (3.13 ± 0.83), which looks almost like grade 3 on the Goutallier scale (Figure 8). At both eight weeks and 12 weeks, the average fat grading was (3.88 ± 0.35) which was consistent with grade 4 of the Goutallier classification system (Figures 9 and 10). These average values were compared to the sham group (0.5 ± 0.53). However, there was a significant difference in fat grades (*p* < 0.05) between sham group and all the operated groups, no significant differences between the two- and four-week groups and between eight- and 12-week groups (Figure 11).

### 3.4. Grading of Muscle Atrophy

**Figure 12:**
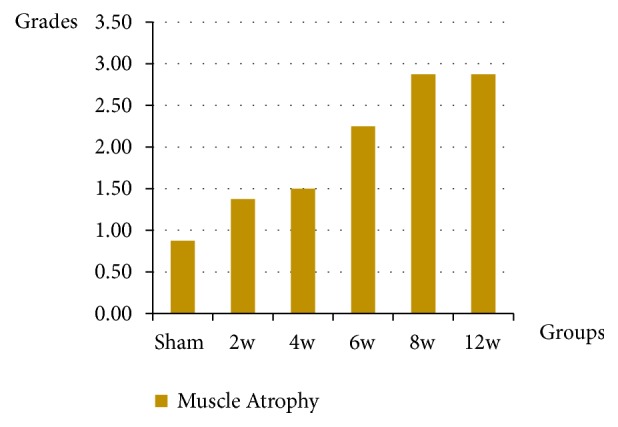
The graph shows the muscle atrophy grading and the data represented by mean ± SD.

The muscle atrophy was graded based on the angular change of the rounded muscle fiber, centralized myonuclei. The degree of muscle atrophy was (1.38 ± 0.51), (1.50 ± 0.53), and (2.25 ± 0.70) at two, four, and six weeks, respectively, compared to sham group (0.88 ± 0.64). So, muscle atrophy was increased from two weeks to six weeks, ranged from mild to moderate, while an advanced, severe atrophy stage was evident in both the eight-week and 12-week groups with atrophy value (2.88 ± 0.35). However, there was a significant difference (*p* < 0.05) between the sham group and the operated groups for atrophy except within two-week group. There is no significant difference between the two-week group and the four-week group or between the eight-week group and the 12-week group (Figure 12).

### 3.5. Grading of Inflammatory Cells

**Figure 13:**
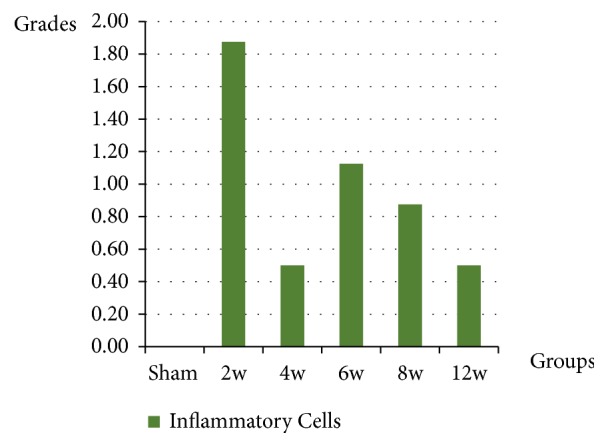
The graph shows the inflammatory cells grading and the data represented by mean ± SD.

The inflammatory process scattered among the muscle fibers appeared as noticeably as two phases. On the one hand, at two weeks, it showed a dramatic increase of inflammatory cells scattered among the muscle fibers with grading value (1.88 ± 0.35). However, in the four-week group, it showed a decline (0.50 ± 0.53). On the other hand, at six weeks, the number of inflammatory cells reappeared to increase with grading value (1.13 ± 0.83). Then, this was followed by a gradual decrease at eight weeks (0.88 ± 0.83) and at 12 weeks (0.50 ± 0.92), in all operated groups (Figure 13).

## 4. Discussion

The objective of this study was to determine the degree of fatty degeneration of the supraspinatus muscle at different time points in New Zealand white adult rabbits and to demonstrate the repairable RCT model in correlation with fatty infiltration grading. We hypothesized that the fatty degeneration of the supraspinatus muscle of the rabbit after detachment of supraspinatus tendon from the footprint would progress gradually with the lapse of time and we could find out the proper time frame which corresponds to the repairable RCT model.

The scar tissue was observed to fill the space between tendon and bone. Adhesion was seen surrounding detached tendon stump. This may cause load transfer into the superficial muscle portion of the supraspinatus muscle. However, Barton et al. [42] have described that the reversal of muscle change is more pronounced in a rat model. It may be partially explained by altered loading environment of the muscle resulting from scar tissue adhesion to the acromion, humerus, and other surrounding structures in the rat model. Scar tissue adhesions to surroundings may play a crucial role in muscle loading following tendon detachment.

In the present study, we observed the majority of samples showed the fatty infiltration in the area below the tangent line among the 40 samples. We considered the area lying below the tangent line, from the superior border of the coracoid to the superior border of the scapular spine, is the area of interest which represents the deep portion of the supraspinatus muscle of the rabbit, corresponding to a binary method of measuring the supraspinatus by Zanetti [43].

The optimal time allowed for development of pathological changes in the rabbit rotator cuff model is controversial. Much research has been done on the process of RCT in rabbits, with many variations in the documented time point [19, 29–31, 34, 44, 45]. The most important findings of the present study were that detachment of the supraspinatus tendon of the rabbit leads to fatty infiltration which was scattered among the muscle fibers in a manner corresponding to Goutallier stage 2 which might be consistent with the repairable RCT model and increased over time.

Barton et al. and others reported that muscle atrophied until four weeks, and muscle fibers were decreased in size. The rapid decline in muscle mass after tendon detachment shows that the muscle is a very sensitive indicator of the state of tendon attachment [42, 46]. In sheep, fat accumulation was also detectable on MRI scans after six weeks, indicating the turning point for fat accumulation is between two and six weeks after tendon release [47]. In our study, based on Goutallier classification, we have found that the two- and four-week groups were consistent with grade 2 fatty infiltrations. The amount of fatty infiltration seen histologically is similar to that reported for human tissue samples of rotator cuff tears with fatty infiltration [4]. It was significantly higher in both the eight-week and 12-week groups than in the two-, four-, and six-week groups. Each group was significantly different from the sham group at each time point.

In the present study, these findings were analyzed in accordance with histological findings. The histological findings were observed at two weeks and showed the appearance of immature adipocytes with large cells—multiple fat vacuoles that indent the nucleus. This occurs in the early stage of adipocytes production. These cells were noticed surrounding the atrophied muscle fibers, and they became mature adipocytes at around four weeks. The inflammatory cells can be seen among the degenerated muscle fibers.

Some of the previous studies showed that fatty infiltration into muscle was increased significantly at the 6-week time point in a manner similar to that found in humans [13, 28, 48–50]. Fabis et al. have demonstrated the development of fatty infiltration in the supraspinatus muscle at six weeks, with contractility changes after supraspinatus tendon tear in a rabbit model. Grade 1 was stable and there was no progression after 6 weeks on CT or histopathology (morphometry). Grade 2 was characterized by morphometric progression of fatty infiltration from 13% at 6 weeks to 21% at 12 weeks. There was no significant progression after 24 weeks [15, 16].

Rowshan et al. described minimal fat accumulation at two weeks after injury, and a significant increase in fat content in chronically detached muscles at six weeks [18]. In the present study, we showed that at six weeks, the fat amount was increased as grade >3. Kim et al. found that after 16 weeks, there was a higher degree of fatty degeneration than there was after eight weeks in rodents model [41]. In our study, we showed that the amount of fat is evident at eight weeks. In addition, fibrosis was observed in the atrophic muscle tissue, and the fat amount corresponded to grade 4 on Goutallier scale.

Muscle atrophy is a critical prognostic factor in determining the outcome of the RCT repair [51]. A problem-free and reliable direct repair of the torn tendon may be predicted by grades 1 and 2. On the other hand, grade 3 atrophy is a predictor of surgical difficulties and the unreliability of a direct suture [10]. Although some authors reported that muscle atrophy and fatty degeneration between muscle fascicles developed after tendon injury progresses over time [16, 29], Buchmann et al. [52] have said that, in a rat model, the level of atrophy showed a peak in the early group (three weeks) and decreased with time. Barton et al. [42] reported that in a rat model, initial loss of muscle mass after tenotomy returned to the control levels at 12 weeks after surgery.

Fabis et al. [15] have described the morphometric changes of the supraspinatus within the middle part of the muscle at 6, 12, and 24 weeks and demonstrated that the size of the rabbit supraspinatus tenotomy is the primary factor affecting the increase of interstitium volume, with maximum effect at 6 weeks after a small tenotomy, although it is increased more after a large tenotomy during 12 weeks of observation. Fabis et al. [29] have also used Goutallier grading system of fatty degeneration with regard to CT examination and observed that rabbit supraspinatus muscle cross-sectional area is decreased by about 50% after the large tenotomy. This was supplemented by the measurement of muscle fibers types I and II diameter as well as interstitium volume increase. Furthermore, Fabis et al. [29] noted the 21% increase of interstitium volume while Gayton et al. [36] in a similar model showed 19% after 12 weeks of observation from rabbit supraspinatus tendon detachment. In our study, we found progress of atrophy in all groups from four weeks to twelve weeks. Mild to moderate atrophy was seen in both four- and six-week groups while extensive atrophy of the supraspinatus muscle was found in the extra muscular area in both eight- and 12-week groups. This is consistent with the ability for RCT repair at four weeks and maybe at six weeks in the rabbit RCT model. In severe atrophy, the affected muscle becomes irreparable. Thus, we agree with previous studies showing that tendons become irreparable to their anatomic footprint due to advanced muscle atrophy [50, 53].

A huge inflammatory infiltrate is recruited after muscle injury, and it is likely to participate in the regulation of muscle regeneration [54–56]. The inflammation was defined as inflammatory cells infiltrate into a tissue, which may be beneficial for tissue repair as it has been demonstrated in several recent studies [57, 58]. Chazaud et al. mentioned that after skeletal muscle injury, the regeneration is characterized by two distinct subsequent phases, each associated with different types of inflammatory cells. Just after injury, recruited macrophages are activated and phagocytose the tissue debris, while preventing myogenic differentiation too early in the repair process [54]. As has been shown to happen in other tissues, the macrophages then switch their phenotype to resolve inflammation [59]. The second phase of muscle repair is characterized by the presence of anti-inflammatory macrophages that directly support myogenesis and myofibers growth [60]. We found the inflammatory cells appearance had two phases. It initially increased at two weeks, and it dropped down at four weeks. This may be due to the early acute tissue reaction after the tendon tear. Then there was a second abrupt increase, at six weeks which may be because of the chronic inflammatory cells recruitment. This was followed by a gradual decrease crossing the eight- and 12-week groups. These observations indicate that the inflammatory process diminishes with time and the potential for the tendon tear to heal by means of resolution and regeneration, and also repair diminishes over time.

Our study has some important limitations. The histologic analysis was semiquantitative. To minimize the potential for bias, three investigators (a musculoskeletal pathologist and two orthopaedic surgeons) independently reviewed the histology specimens, blinded to groups and time points. In the present study, we used four-month-old rabbits, and it is unclear how the development of fatty infiltration would be affected by aging. Because of RCT and fatty infiltration, patients are mostly elderly; therefore, the results of animal model studies should be interpreted with caution. Although fatty degeneration relates directly to muscle function, a functional assessment of the muscle was not performed. To determine the origin of perimuscular fat, relationship between rotator cuff degeneration, and contractile ability, further research is necessary. Also, in this rabbit experiment, spontaneous healing probably resulted in a reversal of atrophy, and the adhesions affect the muscle area above the tangent line which is not consistent exactly with the human condition. Furthermore, this rabbit model does not undergo the robust fibrofatty infiltration that is observed in humans with chronic rotator cuff tears, nor in large-animal rotator cuff chronic injury models [61–63]. The inflammatory process needs more specific focus to determine what kind of cells there are at the early and late stage of the study.

## 5. Conclusion

This study provides histopathologic grading system of fatty infiltration relevant to Goutallier classification system. We have found different fatty infiltration grades at different time points in the rabbit RCT model. At two and four weeks, both fat distribution in rabbit supraspinatus muscles and Goutallier grading scale are mostly similar at grade two. Therefore, we can consider four weeks to be a suitable period for making a repairable RCT animal model for the human research, considering the early acute tissue reaction at 2 weeks after the tendon tears. On the other hand, at eight weeks and 12 weeks, there is advanced fatty infiltrations measured as grade four, with severe muscle atrophy, which may be considered as an irreparable rotator cuff tear model.

## Figures and Tables

**Table 1:** Shows the fatty infiltration distribution.

Pattern of fatty infiltration	Groups				
	2weeks	4weeks	6weeks	8weeks	12weeks
Below tangent line	8/8 (100%)	7/8 (87.5%)	6/8 (75%)	5/8 (62.5%)	1/8 (12.5%)
Above tangent line		1			1
Below and above tangent line			2	3	6
Total	8	8	8	8	8

**Table 2:** Histology grading findings in the central portion of the supraspinatus muscle following tendon release.

Groups	Fat Infiltration		Muscle Atrophy		Inflammatory Cells	
Sham	0.50	±0.53	0.88	±064	0.00	0.00
2w	2.13	±0.64	1.38	±0.51	1.88	±0.35
4w	2.13	±0.35	1.50	±0.53	0.50	±0.53
6w	3.13	±0.83	2.25	±0.70	1.13	±0.83
8w	3.88	±0.35	2.88	±0.35	0.88	±0.83
12w	3.88	±0.35	2.88	±0.35	0.50	±0.92

**Table 3:** Fatty infiltration grading in each group.

Fatty infiltration grading	Groups				
	2 weeks	4 weeks	6 weeks	8 weeks	12 weeks
Grade 1	1				
Grade 2	5	7	2		
Grade 3	2	1	3	1	1
Grade 4			3	7	7
Total	8	8	8	8	8

**Table 4:** Interclass correlation coefficients among the three raters for histological evaluation of fat infiltration, muscle atrophy, and inflammation.

	Fat Infiltration	Muscle Atrophy	Inflammatory Cells
Interclass correlation coefficient	0.95	0.92	0.96
95% CI	(0.92 to 0.97)	(0.87 to 0.95)	(0.94 to 0.98)

## Data Availability

The data used to support the findings of this study are available from the corresponding author upon request.
